# Testing for minimal residual disease in adults with acute lymphoblastic leukemia in Europe: a clinician survey

**DOI:** 10.1186/s12885-018-5002-5

**Published:** 2018-11-12

**Authors:** Arnaud Pigneux, Pau Montesinos, Ze Cong, Xinke Zhang, Anja K. Pownell, Heather Wieffer, Jan McKendrick, Monika Brüggemann

**Affiliations:** 1 Service d’Hématologie Clinique et Thérapie Cellulaire, Univ.Bordeaux, INSERM U035, F-33000 Bordeaux, France; 2 Hematology Department (Torre F, planta 7), Hospital Universitario La Fe, Avinguda Fernando Abril Martorell, 106 CP, 46026 Valencia, Spain; 3 CIBERONC, Instituto Carlos III, Madrid, Spain; 4 Amgen Inc, 1 Amgen Center Dr, Thousand Oaks, CA 91320 USA; 5 PRMA Consulting, Linea House, Harvest Crescent, Ancells Business Park, Fleet, Hampshire, GU51 2UZ UK; 6 Faculty of Health, University of Technology Sydney, Sydney, NSW Australia; 7 Sektion für Hämatologische Spezialdiagnostik, Klinik für Innere Medizin II, Universitätsklinikum Schleswig-Holstein, Campus Kiel, Langer Segen 8-10, D-24105 Kiel, Germany

**Keywords:** MRD testing, ALL, Minimal residual disease, Cross-sectional survey

## Abstract

**Background:**

In acute lymphoblastic leukemia (ALL), the presence of minimal residual disease (MRD) after induction/consolidation chemotherapy is a strong prognostic factor for subsequent relapse and mortality. Accordingly, European clinical guidelines and protocols recommend testing patients who achieve a complete hematological remission (CR) for MRD for the purpose of risk stratification. The aim of this study was to provide quantitative information regarding real-world clinical practice for MRD testing in five European countries.

**Methods:**

A web-based survey was conducted in March/April 2017 in France, Germany, Italy, Spain, and the UK. The survey was developed after consultation with specialist clinicians and a review of published literature. Eligible clinicians (20 per country; 23 in Spain) were board-certified in hemato-oncology or hematology, had at least five years’ experience in their current role after training, had treated at least two patients with B-cell precursor ALL in the 12 months before the survey or at least five patients in the last five years, and had experience of testing for MRD in clinical practice.

**Results:**

MRD testing is now standard practice in the treatment of adult ALL across the five European countries, with common use of recent treatment protocols which specify testing. Respondents estimated that, among clinicians in their country who conduct MRD testing, 73% of patients in first CR (CR1) and 63% of patients in second or later CR (CR2+) are tested for MRD. The median time point reported as most commonly used for the first MRD test, to establish risk status and to determine a treatment plan was four weeks after the start of induction therapy. The timing and frequency of tests is similar across countries. An average of four or five post-CR1 tests per patient in the 12 months after the first MRD test were reported across countries.

**Conclusions:**

This comprehensive study of MRD testing patterns shows consistent practice across France, Germany, Italy, Spain, and the UK with respect to the timing and frequency of MRD testing, aligning with use of national protocols. MRD testing is used in clinical practice also in patients who reach CR2 + .

**Electronic supplementary material:**

The online version of this article (10.1186/s12885-018-5002-5) contains supplementary material, which is available to authorized users.

## Background

Acute lymphoblastic leukemia (ALL) is a rare hematological malignancy in adults, with an estimated incidence of 0.62 per 100,000 adult population [1]. Approximately three-quarters of adult ALL cases are of B-cell lineage, the majority originating from immature B-lymphocytes (B-precursor ALL), with or without the Philadelphia chromosome (Ph) translocation (Ph + or Ph−) [2]. After front-line chemotherapy, the majority of adults with ALL achieve hematological complete remission (CR, defined as < 5% lymphoid blasts in the bone marrow based on a morphologic assessment) [3]. However, leukemic cells may remain in the bone marrow below the threshold of detection of conventional morphologic methods; this is termed minimal residual disease (MRD) [4].

MRD testing uses biomarkers of malignant cells to identify trace levels in bone marrow samples from patients who have achieved CR, using flow cytometry or polymerase chain reaction (PCR)-based techniques [5]. Large prospective multicenter studies have found that 33–47% of patients have MRD after induction therapy [6–9]. Large-scale studies have shown a strong association between the presence of MRD after front-line therapy and poor long-term outcomes, including an increased risk of relapse and shorter relapse-free, disease-free, and overall survival [6, 8, 10, 11]. An analysis of studies by the German Multicenter Study Group for Adult ALL indicated that the five year overall survival rate for patients in CR with MRD after consolidation therapy was 42%, compared with 80% for patients in CR without MRD [8]. A meta-analysis of the association of MRD with clinical outcomes reported 64% of adult patients without MRD were estimated to be event-free at 10 years compared with 21% with MRD. Similarly, the absence of MRD was associated with a 72% reduction in the risk of death; 10-year survival was estimated as 60% compared with 15% [12]. Allogeneic stem cell transplantation (SCT) is an option for patients in hematological CR with MRD but these patients have at least a 70% higher risk of both hematological relapse and death 3 years after SCT than patients without MRD [13–16].

European clinical guidelines and protocols recommend testing adult patients with ALL in first CR (CR1) for MRD to inform risk stratification which drives treatment decisions [17–22]; however, it is unclear to what extent this is reflected in clinical practice. The rationale for this study was to provide quantitative, up-to-date information to describe real-world clinical practice for MRD testing in five European countries (France, Germany, Italy, Spain, and the UK) in adult patients with B-precursor ALL. The objectives were to determine the treatment practices of respondents with respect to use of treatment protocols, timing of MRD tests, methods of testing, frequency of testing, and how the outcomes of MRD testing influence treatment decisions for patients, with both Ph − and Ph + disease.

## Methods

A cross-sectional web-based survey of clinicians who specialize in the treatment of adults with ALL was conducted over an eight week period in March/April 2017 (an initial pilot survey was conducted in January 2017). The Human Research Ethics Committee of the University of Technology, Sydney, Australia, granted ethics approval, and all participants gave written informed consent before starting the survey. Further details on the methodology of the web-based survey are reported in line with the Checklist for Reporting Results of Internet E-Surveys (CHERRIES) [23] (Additional file 1).

### Questionnaire development

The content of the questionnaire was informed by a review of clinical guidelines, treatment protocols for ALL, and publications [24–32]. The draft questionnaire was reviewed by an advisory panel comprising one clinical expert in the use of MRD testing in ALL from each of the five countries. The questionnaire mainly comprised questions with categorical answers and numerical responses, with some follow-up questions requesting free-text responses. The questionnaire included the following topics: participant background, use of treatment protocols (defined in the survey as a published set of rules, often endorsed by a research institution or a clinical body, such as a hematology group), proportion of patients tested for MRD, marker identification for later MRD testing, details of MRD testing in front-line treatment and use of MRD testing in patients with prior relapse. With respect to the MRD testing in front-line treatment, details were asked about the timing and methodology of the “prognostic” MRD test (defined in the survey as the MRD test used to establish risk status and to determine a treatment plan) and subsequent MRD tests. Respondents were asked to report MRD testing patterns according to the Ph+/Ph − status of patients. The questionnaire was programmed for online use and translated into local languages. The questionnaire is included as Additional file 2.

### Participant recruitment

Study participants were recruited by an external medical fieldwork agency from a panel of clinicians who had agreed to participate in such research. Eligible participants, for both the pilot and the main phase study, were board-certified in hemato-oncology or hematology; had at least five years’ experience in their current role after training; had treated at least two patients with B-precursor ALL in the 12 months before the survey, or at least five such patients in the last five years, and had experience of conducting MRD testing in clinical practice. An initial e-mail invitation was sent to clinicians explaining the objective of the study, and a screener questionnaire then followed to determine the clinicians’ eligibility to participate in the survey. Eligible participants were compensated for their time (at the fair market value).

### Pilot study

In order to confirm that the questionnaire was easily understood, well targeted and that it provided informative results, a pilot phase with a draft version of the questionnaire preceded the main survey. Two participants were recruited per country from the same pool of clinicians subsequently approached in the main phase of the survey. An additional eligibility criterion was applied exclusively for the pilot phase, but was removed given concerns over misunderstanding and unwarranted exclusion of potential participants: that participants had to practice in an institution that participated in research into the treatment of adult patients with ALL conducted by one of the European ALL study groups, or that participated in other recent registered clinical trials. Responses were reviewed and a follow-up telephone interview was conducted to collect feedback, which was used to refine the questionnaire for the main survey. Pilot phase data were not included in the survey results, and clinicians who participated in the pilot phase were not approached again for the main survey.

### Survey conduct

The web-based survey was completed by eligible participants without time restrictions and in the local language. The survey was designed to take around 45 min to complete.

### Statistical analysis

The survey was a descriptive study, and no formal statistical hypotheses were tested. All data were analyzed separately for each country according to a pre-specified analysis plan and pooled analyses across all countries were also conducted. The analyses were performed using SAS statistical software, version 9.4. Continuous variables were summarized in the form of mean and standard deviation (SD) and median and interquartile range (IQR); both parametric and non-parametric approaches were used because the mean value may be sensitive to a few outlying observations due to the small sample size. For categorical variables, the number and percentage of respondents was calculated for each category. Free-text fields were reviewed and categorized.

## Results

### Demographics and treatment practice of study respondents

Twenty clinicians per country completed the survey, except for Spain where 23 clinicians participated. Across countries respondents had a mean of 16 years’ experience in treating ALL since completion of specialty training. Most respondents reported that their institution was a university hospital (66%, *n* = 68), and 78% (*n* = 80) reported that their institution participated in research conducted by a European ALL study group or in other registered trials (see Additional file 3: Table S1).

Across all five countries, respondents reported that a mean of 70% (SD 36%; ranging from 50% in the UK to 89% in Spain) of their adult patients receiving front-line therapy for B-precursor Ph– ALL had their treatment pathway determined by a protocol. For patients with Ph + ALL, a mean of 73% (SD 35%; ranging from 61% in the UK to 88% in Spain) of patients in the respondents’ caseloads were reported to have their treatment determined by a protocol. Other patients were reported to have their treatment determined by an investigational clinical trial protocol (mean [SD]; 18% [30%] for Ph − ALL and 14% [25%] for Ph + ALL across countries), or by neither of these (mean [SD] 12% [24%] for Ph-ALL and 13% [28%] for Ph + ALL across countries). In the UK, the proportion of patients whose treatment was determined by an investigational clinical trial protocol were considerably higher than that across the rest of the participating countries (Ph- 40% vs Ph + 23%). This may be due to inconsistencies in whether respondents categorized the UK ALL 14 study [22] as a protocol or investigational clinical trial. Respondents selected recent protocols in their country as their most common choice to guide front-line treatment; these protocols require MRD testing (Table 1). The time points for MRD testing and key recommendations from the protocols are provided in the supplementary data (see Additional file 3: Table S3).

**Table 1:** Protocols selected as the most common choice

	Protocol/trial (% of responding clinicians selecting protocol/trial as most common choice)			
	Adults with Ph − disease		Adults with Ph + disease	
France	GRAALL 2014	50	ALL GRAALLPHAG06/EWALL-PH–01	35
Germany	GMALL 08 2013	65	GMALL 08 2013	65
Italy^a^	GIMEMA LAL1913	42	GIMEMA LAL1811	25
Spain	PETHEMA LAL-AR/2011	57	PETHEMA LAL PH–2008	59
UK	UK ALL 14	70	UK ALL 14	68

^a^Use of a range of GIMEMA protocols was reported for Italy, reflecting different populations (GIMEMA LAL1913, GIMEMA LAL1104, and GIMEMA LAL1308 for Ph − disease; GIMEMA LAL1811 and GIMEMA LAL1408 for Ph + disease)

ALL, acute lymphoblastic leukemia; Ph, Philadelphia chromosome translocation

### Use of MRD testing

**Fig. 1:**
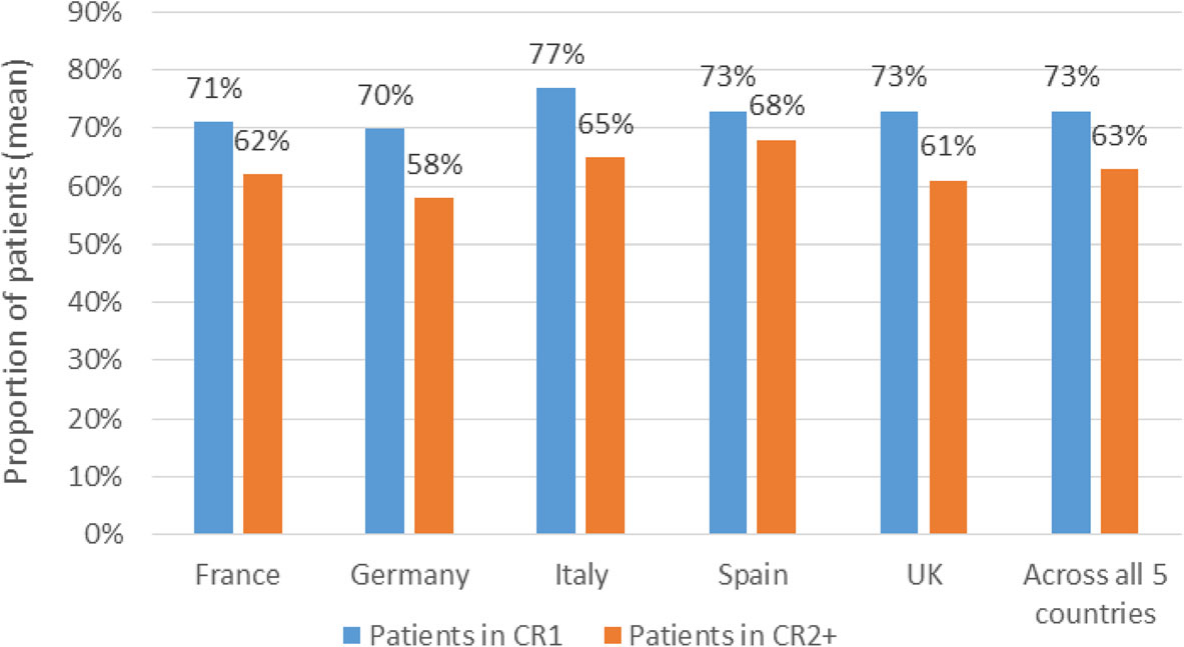
Estimated proportion of adults with B-precursor ALL tested for MRD amongst clinicians who test for MRD. Footnote: ALL, acute lymphoblastic leukemia; CR1, first complete remission; CR2+, second complete remission or later; MRD, minimal residual disease

Respondents were asked to estimate the proportion of patients tested for MRD in their country amongst adults with B-precursor ALL. Across countries, it was estimated that amongst clinicians who test for MRD, 73% (SD 24%) of patients in CR1 and 63% (SD 27%) in CR2+ are tested for MRD (Figure 1). These results indicate that MRD testing is common in CR2+ as well as in CR1, although at a slightly lower rate than in CR1.

### Testing in newly diagnosed patients

Marker identification for later MRD testing was reported as usual practice by the majority of surveyed clinicians. Across countries, 78% of respondents requested marker identification for later MRD testing for all their patients with Ph − disease receiving front-line treatment (ranging from 70% respondents in France to 90% in the UK); the corresponding proportion for patients with Ph + disease was 83% (ranging from 71% in Germany to 90% in Italy). Respondents who did not report requesting marker identification for all their patients most frequently indicated the poor physical status of the patient as the reason.

### Timing of the first prognostic MRD test and methods of testing

**Fig. 2:**
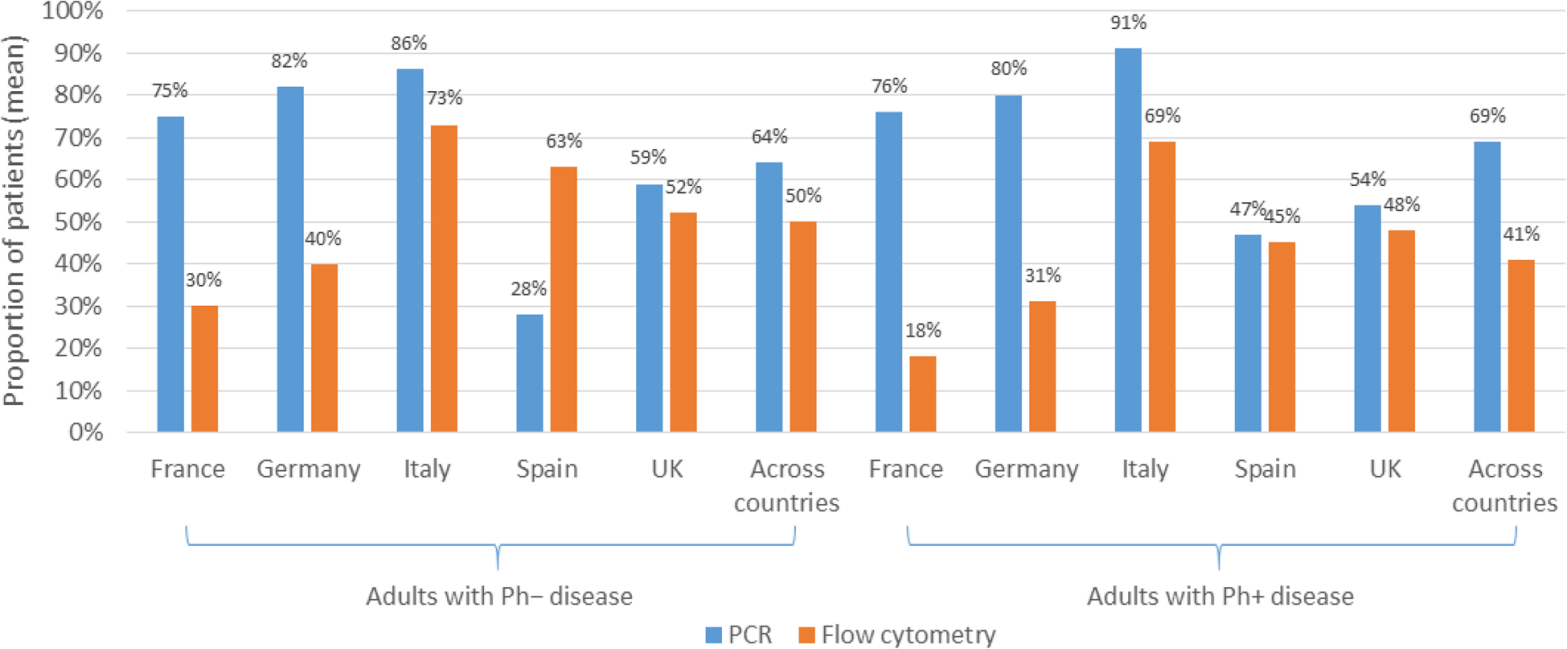
Method used for MRD testing for patients in CR1 for prognostic MRD test. Footnote: CR1, first complete remission, MRD, minimal residual disease; PCR, polymerase chain reaction; Ph, Philadelphia chromosome translocation

Across countries, the median time point reported as most commonly used for conducting the first “prognostic” MRD test was 4 weeks (IQR 4–8 weeks) after the start of induction therapy, for both Ph − and Ph + disease (see Additional file 3: Table S4). Across countries, clinicians reported that they requested prognostic MRD tests based on PCR for 64% (SD 40%) and 69% (SD 40%) of their patients in CR1 with Ph − and Ph + disease, respectively. The corresponding figures for MRD tests based on flow cytometry were 50% (SD 43%) and 41% (SD 44%), respectively (Figure 2). The results indicated that some clinicians use more than one method of testing. A similar mix of technologies were used for Ph − and Ph + disease in all countries except in Spain, where use of flow cytometry predominated for Ph − disease. Clinicians were further asked whether this test was conducted at a central laboratory, and/or a local laboratory. For patients in CR1 with Ph − disease, the majority of clinicians in all countries reported using central laboratories for testing, with or without local laboratory testing in addition, ranging from 89% in France to 52% of respondents in Spain. For patients in CR1 with Ph + disease, the majority of respondents reported using central laboratories, with or without local laboratory testing, in all countries except Spain (see Additional file 3: Table S5).

### Treatment decisions based on prognostic test results

For patients with MRD− status determined by the prognostic test, the most commonly reported treatment decision guided by this result was to start maintenance or consolidation treatment (selected by 82% of respondents for Ph − disease [ranging from 72% in France to 92% in Italy] and 72% respondents for Ph + disease [ranging from 61% in the UK to 78% in France]). Across countries, 28 and 41% respondents (Ph − and Ph + disease, respectively) stated that MRD− status in the prognostic test would influence the decision on suitability for SCT, and 26 and 30% reported that this would indicate the start of treatment intensification (see Additional file 3: Table S6).

For patients with MRD+ status determined by the prognostic test, the most commonly reported treatment decisions were to start treatment intensification (67% of respondents for Ph − and 61% for Ph + disease) and to decide suitability for SCT (59% respondents for Ph − and 62% for Ph + disease), both aligning with recommendations that MRD+ status should be considered an indication for more intensive treatment. MRD+ status was reported to indicate the start of maintenance or consolidation treatment by less than one third of the respondents (see Additional file 3: Table S6).

### Frequency of post-CR MRD testing

Clinicians reported that they most commonly conducted an average of four or five tests per patient in the 12 months after the prognostic test (Table 2). The average number of tests was reported to be lower in patients with MRD+ than with MRD− status in most countries (Table 2). The mean frequency of testing across countries was every four months in patients with Ph − disease and MRD+ status, every two months in patients with Ph + disease and MRD− status, and every three months in other groups (data not shown).

**Table 2:** Most common number of post-CR tests over 12 months after the prognostic MRD test

	Adults with Ph − disease				Adults with Ph + disease			
	MRD−		MRD+		MRD−		MRD+	
	Mean (SD)	Median (IQR)	Mean (SD)	Median (IQR)	Mean (SD)	Median (IQR)	Mean (SD)	Median (IQR)
France	3 (3)	3 (2–3)	3 (2)	2 (2–3)	4 (5)	3 (2–4)	3 (4)	2 (2–3)
Germany	5 (5)	3 (2–4)	4 (4)	2 (2–6)	5 (5)	3 (2–4)	4 (5)	3 (2–5)
Italy	6 (6)	4 (3–7)	6 (4)	4 (3–6)	6 (6)	4 (3–6)	5 (6)	4 (2–6)
Spain	5 (2)	5 (3–6)	4 (2)	3 (3–6)	5 (2)	5 (3–6)	4 (2)	3 (3–6)
UK	6 (5)	4 (2–8)	5 (5)	3 (2–6)	5 (4)	4 (3–8)	6 (5)	4 (3–7)
Across countries	5 (4)	4 (2–6)	4 (4)	3 (2–5)	5 (4)	4 (3–6)	5 (5)	3 (2–5)

CR, complete remission, MRD, minimal residual disease; Ph, Philadelphia chromosome translocation; SD, standard deviation; IQR, interquartile range

### Testing in patients in CR2 and later

Across countries, 86% of respondents (ranging from 80% in the UK and Italy, to 100% in France) indicated that they had conducted MRD testing in patients in CR2+ in the last 12 months; these clinicians were asked about the reasons, and the frequency of MRD testing in these patients. The most commonly reported reasons in patients with prior relapse were to monitor for progression (26% of respondents), to offer additional therapeutic options (22%), and to inform decision-making on SCT (19%). The median frequency of testing was reported to be every three months (IQR 2–3) across countries.

## Discussion

To our knowledge, this is the first comprehensive study of patterns of MRD testing in adult ALL in Europe. The survey has shown that MRD testing is now standard practice in the treatment of adult ALL across the five European countries included. This is consistent with the common use in these countries, as identified in this survey, of recent national protocols that recommend MRD testing to inform treatment decisions. The results suggest that only a small proportion of patients are not tested for MRD on achieving CR1, which is likely to be due to individual clinical reasons such as poor physical status (the most common reason provided in the survey). MRD testing practices are broadly similar across the countries in terms of timing, frequency, and technology of tests, reflecting broad alignment between national protocols, except in Spain, where flow cytometry rather than PCR was reported as more commonly used for patients with Ph − disease.

However, the survey identified small differences in the number of MRD tests conducted in patient groups based on their initial MRD status in clinical practice: the lower number of tests in patients with MRD+ status may reflect the use of SCT in these patients, with the frequency of MRD testing after SCT not specified by protocols and potentially less; alternatively it may reflect poorer survival in these patients.

The survey identified that MRD testing in clinical practice is commonly used in patients in CR2+ as well as in CR1. This was somewhat unexpected given that the treatment pathway for patients with prior relapse is not driven by standard protocols in most countries, hence MRD testing in that setting is not protocol driven. In Spain, the PETHEMA group has published a protocol for the treatment of relapsed ALL that recommends MRD testing for younger patients who will undergo SCT: the results of this survey suggest that there is high use of this protocol. The survey suggests that MRD status is of interest for clinicians treating relapsed ALL, although our results indicate that the clinical rationale and implications of this vary between clinicians (MRD status was reported to be used for prognosis, monitoring, or to inform treatment decisions).

As well as being the first study to comprehensively assess MRD testing patterns in Europe, this study has several other strengths. The methods for survey development were robust, involving a thorough review of guidelines and the literature, as well as seeking input from clinicians. A pilot survey was conducted to ensure the validity of the survey. The number of participants was pre-specified for each country, to ensure the data collected were representative and accurate. Finally, participants had to meet stringent inclusion criteria to ensure they had extensive experience of treating B-precursor ALL and MRD testing in clinical practice.

The study also had some potential limitations. The results presented here were based on clinicians’ estimates, not patient-level observations. Respondents were recruited through a medical fieldwork agency, from a pool of clinicians who had previously indicated their willingness to participate in online research; this pool may not be representative of all clinicians who treat ALL in each country. However, given the limited number of clinicians treating adult patients with ALL in each country, we are confident that with our sample size, our results can reflect the national practice patterns in general. This survey also only included clinicians in five larger Western European countries: local protocols and availability of testing technologies may differ elsewhere and mean these results may not reflect European clinical practice as a whole.

Further we have observed some results that suggest misinterpretation of specific questions.

- The current Spanish protocols (from PETHEMA), and known availability of testing technologies in this country, means that we would expect the vast majority of clinicians in Spain treating ALL to report use of flow cytometry for their patients with Ph– disease; the results showed that flow cytometry indeed was more commonly used, but an average of 28% of respondents’ caseloads of Ph– patients were reported to have samples tested by PCR; this was higher than expected. For Ph + disease, PCR-based detection of the characteristic *BCR-ABL* fusion gene had been expected to be used for all patients in line with protocols, but flow cytometry was reported to be used for 45% of respondents’ caseloads.
- A minority of respondents provided illogical responses when asked about the treatment decisions following a negative prognostic MRD test (respondents reported that they would intensify treatment in 26% of patients with Ph– and 30% of patients with Ph + disease): as treatment intensification would not be a logical consequence of this result, we assume that these respondents misinterpreted the question.

Such limitations may arise from the methodology used – for example, there was no opportunity for dialogue or clarification during or after completion of the survey. Although this avoids potential bias to report ideal rather than actual practice, it also meant that unexpected results could not be investigated further to determine whether they reflected misinterpretations or mistakes in completing the survey. A more open, interview-based approach may be preferable in the future for such a specialized disease area to avoid these issues.

A next step in this research would be to extend the clinical interpretation of the results through focused discussions with practicing clinicians in each country, including identifying where and why differences arise, and when more individualized (at a patient or institution-level) treatment decisions are needed; such discussion could inform the development of future protocols. MRD testing in clinical practice may be sensitive to future changes in technology and treatments, including increased standardization within and across countries as a result of collaborative initiatives and new treatments indicated by MRD status; also if increasing risk stratification of ALL patient populations leads to different risk-based treatment pathways for patient subtypes, e.g., Ph-like ALL. It would be useful to address these issues again at a later date, to increase understanding of the best way to use MRD testing in risk evaluation and treatment decision-making.

## Conclusions

This study provides estimates of the extent of MRD testing and protocol use in France, Germany, Italy, Spain, and the UK. The timing and frequency of MRD testing in clinical practice is consistent across the countries, as is the use of MRD testing outcomes to guide treatments decisions. The prevalence of testing differs between patients with MRD+ and MRD− status, with fewer tests in the former group.

## Additional files

Additional file 1: CHERRIES checklist for web surveys. (DOCX 29 kb)

Additional file 2: Abbreviated version of the questionnaire. (DOCX 115 kb)

Additional file 3: Supplementary tables. (DOCX 48 kb)

## Abbreviations

ALL: Acute lymphoblastic leukemia
CHERRIES: Checklist for Reporting Results of Internet E-Surveys
CR: Complete remission
CR1: First remission
CR2 +: Second or later remission.
IQR: Interquartile range
MRD: Minimal residual disease
PCR: Polymerase chain reaction
Ph: Philadelphia chromosome
SD: Standard deviation
SCT: Stem cell transplantation
