# Supplementary material for: Testing for minimal residual disease in adults with acute lymphoblastic leukemia in Europe: a clinician survey
Source: BMC Cancer. 2018 Nov 12;18:1100. doi: 10.1186/s12885-018-5002-5 (PMC6233570; doi:10.1186/s12885-018-5002-5)
Supplement: Supplementary file 1 — CHERRIES checklist for web surveys. (DOCX 29 kb) [file 12885_2018_5002_MOESM1_ESM.docx]

Additional file 1

CHERRIES checklist

Reference: Eysenbach G. Improving the Quality of Web Surveys: The Checklist for Reporting Results of Internet E-Surveys (CHERRIES). Journal of Medical Internet Research. 2004;6(3):e34. doi:10.2196/jmir.6.3.e34.

| **Item Category** | **Checklist Item** | **Explanation** | **Description** |
| --- | --- | --- | --- |
| Design | Describe survey design | Describe target population, sample frame. Is the sample a convenience sample?  (In “open” surveys this is most likely.) | The target population was clinicians who specialize in the treatment of adults with ALL. Study participants were recruited by an external medical fieldwork agency from a panel of clinicians who had agreed to participate in such research. Eligible participants, for both the pilot and the main phase study, were board-certified in hemato-oncology or hematology; had at least 5 years’ experience in their current role after training; had treated at least two patients with B-precursor ALL in the 12 months before the survey, or at least five such patients in the last 5 years, and had experience of conducting MRD testing in clinical practice. |
| IRB (Institutional Review  Board) approval and informed  consent process | IRB approval | Mention whether the study has been approved by an IRB. | The Human Research Ethics Committee of the University of Technology, Sydney, Australia, granted ethics approval. |
|  | Informed consent | Describe the informed consent process. Where were the participants told the length of time of the survey, which data were stored and where and for how long, who the investigator was, and the purpose of the study? | All participants, as members of a web-based panel, had already provided informed consent to participate in online surveys. Informed consent for the present survey was obtained from all those agreeing to complete a survey, with participants informed on the first page that the survey was understand how clinicians use MRD testing in clinical practice in adult patients with B-precursor ALL, the identity of the investigators, that it would take approximately 45 minutes to complete, and that all responses were confidential and anonymous; also that data would be stored in a secure and de-identified for a period of seven years. Consent was indicated by respondents checking a box on the web-page: this was required for respondents to proceed with the survey. |
|  | Data protection | If any personal information was collected or stored, describe what mechanisms were used to protect unauthorized access. | Standard operating procedures of the external medical fieldwork agency were in place to ensure data protection, including de-identification of respondent data before analysis. The fully de-identified dataset is kept on password protected computers. |
| Development and pre-testing | Development and testing | State how the survey was developed, including whether the usability and technical functionality of the electronic questionnaire had been tested before fielding the questionnaire. | The content of the questionnaire was informed by a review of clinical guidelines, treatment protocols for ALL, and publications. The draft questionnaire was reviewed by an advisory panel comprising one clinical expert in the use of MRD testing in ALL from each of the five countries. In order to confirm that the questionnaire was easily understood, well targeted and that it provided informative results, a pilot phase with a draft version of the questionnaire preceded the main survey.  Testing of the technical functionality of the electronic questionnaire was conducted by the external medical fieldwork agency and by the investigators prior to fielding. |
| Recruitment process and description  of the sample having access  to the questionnaire | Open survey versus closed survey | An “open survey” is a survey open for each visitor of a site, while a closed survey is only open to a sample which the investigator knows (password-protected survey). | This was an open survey: the medical fieldwork agency recruited a cohort of clinicians from their panel of potential survey participants. |
|  | Contact mode | Indicate whether or not the initial contact with the potential participants was made on the Internet. (Investigators may also send out questionnaires by mail and allow for Web-based data entry.) | Invitations to participate in the survey were sent by email to potential participants identified from the medical fieldwork agency panel based on their specialty in hematology/hemato-oncology. |
|  | Advertising the survey | How/where was the survey announced or advertised? Some examples are offline media (newspapers), or online (mailing lists – If yes, which ones?) or banner Advertising the survey ads (Where were these banner ads posted and what did they look like?). It is important to know the wording of the announcement as it will heavily influence who chooses to participate. Ideally the survey announcement should be published as an appendix. | The survey was not advertised: as above, invitations to participate were sent to potential participants. |
| Survey administration | Web/E-mail | State the type of e-survey (e.g., one posted on a Web site, or one sent out through e-mail). If it is an e-mail survey, were the responses entered manually into a database, or was there an automatic method for capturing responses? | This was a web-based survey, with responses captured through the online survey platform. |
|  | Context | Describe the Web site (for mailing list/newsgroup) in which the survey was posted. What is the Web site about, who is visiting it, what are visitors normally looking for? Discuss to what degree the content of the Web site could pre-select the sample or influence the results. For example, a survey about vaccination on an anti-immunization Web site will have different results from a Web survey conducted on a government Web site | NA |
|  | Mandatory/voluntary | Was it a mandatory survey to be filled in by every visitor who wanted to enter the Web site, or was it a voluntary survey? | This was a voluntary survey. |
|  | Incentives | Were any incentives offered (e.g., monetary, prizes, or non-monetary incentives such as an offer to provide the survey results)? | Eligible participants were compensated for their time (at the fair market value). |
|  | Time/Date | In what timeframe were the data collected? | The survey was conducted over an eight week period in March/April 2017 (an initial pilot survey was conducted in January 2017). |
|  | Randomization of items or questionnaires | To prevent biases items can be randomized or alternated. | No randomization was used. |
|  | Adaptive questioning | Use adaptive questioning (certain items, or only conditionally displayed based on responses to other items) to reduce number and complexity of the questions. | Adaptive questioning was used. Relevant survey items were displayed based on previous responses, e.g., only those who indicated that they had conducted MRD testing in CR2+ in the last 12 months were asked further questions about this. |
|  | Number of Items | What was the number of questionnaire items per page? The number of items is an important factor for the completion rate. | Between 1 and 3 questions were displayed per survey page. |
|  | Number of screens (pages) | Over how many pages was the questionnaire distributed? The number of items is an important factor for the completion rate. | The full survey was distributed over approximately 94 pages. |
|  | Completeness check | It is technically possible to do consistency or completeness checks before the questionnaire is submitted. Was this done, and if “yes”, how (usually JAVAScript)? An alternative is to check for completeness after the questionnaire has been submitted (and highlight mandatory items). If this has been done, it should be reported. All items should provide a non-response option such as “not applicable” or “rather not say”, and selection of one response option should be enforced. | Respondents prompted to complete outstanding items before leaving each page and could not proceed until completed. |
|  | Review step | State whether respondents were able to review and change their answers (eg, through a Back button or a Review step which displays a summary of the responses and asks the respondents if they are correct). | Respondents were unable to change their responses once submitted. |
| Response rates | Unique site visitor | If you provide view rates or participation rates, you need to define how you determined a unique visitor. There are different techniques available, based on IP addresses or cookies or both. | Determination of unique visitors was handled by the panel provider, whereby invitations to participate in the study were sent only to unique validated panellist accounts. |
|  | View rate (Ratio of unique survey  visitors/unique site visitors) | Requires counting unique visitors to the first page of the survey, divided by the number of unique site visitors (not page views!). It is not unusual to have view rates of less than 0.1 % if the survey is voluntary | NA – respondents were invited |
|  | Participation rate (Ratio of  unique visitors who agreed to  participate/unique first survey  page visitors) | Count the unique number of people who filled in the first survey page (or agreed  to participate, for example by checking a checkbox), divided by visitors who visit the first page of the survey (or the informed consents page, if present). This can also be called “recruitment” rate. | NA - Participants were direct from the email invitation to the participant information and informed consent which was the first page of the survey |
|  | Completion rate (Ratio of users  who finished the survey/users  who agreed to participate) | The number of people submitting the last questionnaire page, divided by the number of people who agreed to participate (or submitted the first survey page).  This is only relevant if there is a separate “informed consent” page or if the survey goes over several pages. This is a measure for attrition. Note that “completion” can involve leaving questionnaire items blank. This is not a measure for how completely questionnaires were filled in. (If you need a measure for this, use the word “completeness rate”.) | The completion rate (the number who completed the survey/the number who started the survey) was 58%. |
| Preventing multiple entries from  the same individual | Cookies used | Indicate whether cookies were used to assign a unique user identifier to each client computer. If so, mention the page on which the cookie was set and read, and how long the cookie was valid. Were duplicate entries avoided by preventing users access to the survey twice; or were duplicate database entries having the same user ID eliminated before analysis? In the latter case, which entries were kept for analysis (e.g., the first entry or the most recent)? | A device check mechanism was in place to prevent an individual from completing the survey twice from the same device. Each survey link included in the invitation was created to be unique, such that once it is used, it cannot be used again. Further, automated checks for duplicate respondents via personal identifiable information were carried out. |
|  | IP check | Indicate whether the IP address of the client computer was used to identify potential duplicate entries from the same user. If so, mention the period of time for which no two entries from the same IP address were allowed (e.g., 24 hours).  Were duplicate entries avoided by preventing users with the same IP address access to the survey twice; or were duplicate database entries having the same IP address within a given period of time eliminated before analysis? If the latter, which entries were kept for analysis (eg, the first entry or the most recent)? | As above |
|  | Log file analysis | Indicate whether other techniques to analyze the log file for identification of multiple entries were used. If so, please describe. | As above |
|  | Registration | In “closed” (non-open) surveys, users need to login first and it is easier to prevent duplicate entries from the same user. Describe how this was done. For example, was the survey never displayed a second time once the user had filled it in, or was the username stored together with the survey results and later eliminated?  If the latter, which entries were kept for analysis (e.g., the first entry or the most recent)? | As above |
| Analysis | Handling of incomplete questionnaires | Were only completed questionnaires analyzed? Were questionnaires which terminated early (where, for example, users did not go through all questionnaire pages) also analyzed? | Only completed questionnaires were included in the final dataset. |
|  | Questionnaires submitted with  an atypical timestamp | Some investigators may measure the time people needed to fill in a questionnaire and exclude questionnaires that were submitted too soon. Specify the timeframe that was used as a cut-off point, and describe how this point was determined. | Quality checks were in place to review respondents who completed the survey in less than one third of the median time for survey completion. |
|  | Statistical correction | Indicate whether any methods such as weighting of items or propensity scores have been used to adjust for the non-representative sample; if so, please describe the methods. | No adjustments were made. |
