# Supplementary material for: Testing for minimal residual disease in adults with acute lymphoblastic leukemia in Europe: a clinician survey
Source: BMC Cancer. 2018 Nov 12;18:1100. doi: 10.1186/s12885-018-5002-5 (PMC6233570; doi:10.1186/s12885-018-5002-5)
Supplement: Supplementary file 2 — Abbreviated version of the questionnaire. (DOCX 115 kb) [file 12885_2018_5002_MOESM2_ESM.docx]

Additional file 2

**Questionnaire for main phase of survey**

# About you and your clinic

Q1. In which region is your institution based?

| [Radio button] | Region of Paris | Région Parisienne (Île de France) |
| --- | --- | --- |
| [Radio button] | North East | Nord-Est (Nord-Pas-de-Calais, Lorraine, Alsace, Franche-Comté, Bourgogne, Champagne-Ardenne, Picardie) |
| [Radio button] | South East | Sud-Est (Rhône-Alpes, Auvergne, Méditerranée (Languedoc-Roussillon, Provence-Alpes-Côte d'Azur, Corse) |
| [Radio button] | South West | Sud-Ouest (Aquitaine, Midi-Pyrénées, Limousin, Poitou-Charentes) |
| [Radio button] | North West | Nord-Ouest (Haute-Normandie, Centre, Basse-Normandie, Bretagne, Pays de la Loire) |
| [Radio button] | North | **Nord** (Bremen, Hamburg, Mecklenburg-Vorpommern, Niedersachsen, Schleswig-Holstein) |
| [Radio button] | East | **Ost** (Berlin, Brandenburg, Sachsen, Sachsen-Anhalt) |
| [Radio button] | West | **West** (Nordrhein-Westfalen, Rheinland-Pfalz, Saarland) |
| [Radio button] | Central | **Zentral** (Hessen, Thüringen) |
| [Radio button] | South | **Süd** (Baden-Württemberg, Bayern) |
| [Radio button] | North East | **Nord-Est** (Friuli-Venezia Giulia, Veneto, Trentino-Alto Adige, Emilia-Romagna) |
| [Radio button] | North West | **Nord-Ovest** (Valle d'Aosta, Piemonte, Lombardia, Liguria) |
| [Radio button] | Central | **Centro** (Toscana, Marche, Lazio, Umbria) |
| [Radio button] | South | **Sud** (Abruzzo, Molise, Campania, Basilicata, Puglia, Calabria) |
| [Radio button] | Islands | **Isole** (Sicilia, Sardegna) |
| [Radio button] | North East | **Noreste** (Aragón, País Vasco, La Rioja, Comunidad Foral de Navarra) |
| [Radio button] | North West | **Noroeste** (Principado de Asturias, Cantabria, Galicia) |
| [Radio button] | East | **Este** (Islas Baleares, Cataluña, Comunidad Valenciana) |
| [Radio button] | Madrid | **Comunidad de Madrid** |
| [Radio button] | Centre | **Centro** (Castilla y León, Castilla-La Mancha, Extremadura) |
| [Radio button] | South | **Sur** (Andalucía, Ciudad Autónoma de Ceuta, Ciudad Autónoma de Melilla, Región de Murcia) |
| [Radio button] | Canaries | **Islas Canarias** |
| [Radio button] | Scotland | **Scotland** |
| [Radio button] | Northern England | **Northern England** |
| [Radio button] | Northern Ireland | **Northern Ireland** |
| [Radio button] | Wales | **Wales** |
| [Radio button] | Midlands | **Midlands** |
| [Radio button] | London | **London** |
| [Radio button] | South East England | **South East England** |
| [Radio button] | South West England | **South West England** |

Q2. Which type of centre best describes your institution?

*Please select one answer only.*

| [Radio button] | University hospital |
| --- | --- |
| [Radio button] | Community/urban/general hospital |
| [Radio button] | Cancer hospital or specialist oncology centre |
| [Radio button] | Specialist haematology centre |
| [Radio button] | Ambulatory care clinic |
| [Radio button] | Other |

Q2b. Does the institution where you practice participate in research into treatment of adult patients with ALL

- conducted by one of the European ALL study groups (including PETHEMA, GRAALL, GMALL, GIMEMA, NILG, GOELAMS, UK NCRI), or
- other recent registered clinical trials?

| [Radio button] | Yes |
| --- | --- |
| [Radio button] | No |

Q3. How many adults with B-precursor ALL that you have treated in the past 12 months have achieved complete response, either CR1 or CR2 or later (CR2+)? In how many of these patients did you test for MRD?

|  | Adults with Ph− disease | | Adults with Ph+ disease | | Total |
| --- | --- | --- | --- | --- | --- |
|  | Patients in CR1 | Patients in CR2+ | Patients in CR1 | Patients in CR2+ |  |
| **Number of patients treated** |  |  |  |  |  |
| Number of patients who achieved CR |  |  |  |  |  |
| Number of patients in CR where MRD testing was conducted |  |  |  |  |  |

Q4a. Of the clinicians in your country who treat adults with B-precursor ALL, please estimate the proportion who test for MRD.

|  | Patients in CR1 | Patients in CR2+ |
| --- | --- | --- |
| **Proportion of clinicians** | % of clinicians | % of clinicians |

Q4b. For those clinicians who test for MRD, please estimate the extent to which they do this:

|  | Patients in CR1 | Patients in CR2+ |
| --- | --- | --- |
| **Proportion of patients** | % of patients | % of patients |

Q5. Can adults (aged 18 years and over) with B-precursor ALL receive autologous or allogenic SCT while in the care of you or your team?

| [Radio button] | Yes |
| --- | --- |
| [Radio button] | No |

# Treatment protocols and clinical trials for first-line treatment

The following questions explore the treatment protocols (“protocols”) and investigational clinical trials (“trials”) you have followed in the past 12 months for the **first-line** treatment of adults with B-precursor ALL.

- The term “protocol” refers to a published set of rules for the first-line treatment of adults with B-precursor ALL. It is often a protocol endorsed by a research institution or a clinical body, such as a haematology group.
- The term “trial” refers to an investigational clinical trial of a pharmaceutical treatment used in the first-line treatment of adults with B-precursor ALL. It is often a clinical trial sponsored by a pharmaceutical company.
- Examples of protocols and trials are GIMEMA LAL1308, MRC UKALL 60 or PETHEMA LAL Ph-2008 . Please do not consider chemotherapy regimens, such as hyperCVAD, as a protocol for the purpose of this survey.

These questions relate to your caseload of **adults** with **B-precursor ALL** whom you treated in **first line**.

Q6a. Of the patients you have treated in the past 12 months, what determined the treatment pathway they followed?

|  | Adults with Ph− disease | Adults with Ph+ disease |
| --- | --- | --- |
| **Number in caseload** |  |  |
| **The treatment pathway is determined by a protocol** |  |  |
| **The treatment pathway is determined by the investigational clinical trial protocol** |  |  |
| **The treatment pathway is not determined by protocol or trial** |  |  |

Q6b. The treatment pathway for adults with B-precursor ALL can be determined by a protocol or a trial (definition of protocol and trial).

Which protocols or trials have you used to guide the treatment pathway for patients you have treated in first line in the past 12 months? Of those, which one have you followed most commonly?

*For each patient group, please select all that you have used and indicate the one protocol/trial that you most commonly followed in the past 12 months.*

| Treatment protocols | Adults with Ph− disease | | Treatment protocols | Adults with Ph+ disease | |
| --- | --- | --- | --- | --- | --- |
|  | Used | Most common |  | Used | Most common |
|  | [Radio button] | [Radio button] |  | [Radio button] | [Radio button] |
|  | [Radio button] | [Radio button] |  | [Radio button] | [Radio button] |
|  | [Radio button] | [Radio button] |  | [Radio button] | [Radio button] |
|  | [Radio button] | [Radio button] |  | [Radio button] | [Radio button] |
|  | [Radio button] | [Radio button] |  | [Radio button] | [Radio button] |
|  | [Radio button] | [Radio button] |  | [Radio button] | [Radio button] |
| **Other treatment protocol**  Please specify protocol name_________ | [Radio button] | [Radio button] | **Other treatment protocol**  Please specify protocol name_________ | [Radio button] | [Radio button] |
| Investigational clinical trial  Please specify clinical trial name/number  _____________ | [Radio button] | [Radio button] | Investigational clinical trial  Please specify clinical trial name/number  _____________ | [Radio button] | [Radio button] |

Q6c. You have now selected a most commonly used protocol/trial (for patients with Ph− disease and the same for patients with Ph+ disease, if you treat these patients). Please indicate the subgroups represented in the protocol/trial you selected:

*Please select all that apply.*

|  | Adults with Ph− disease | Adults with Ph+ disease |
| --- | --- | --- |
| **Most common protocol/trial** |  |  |
| **All adults** | [Tick box] | [Tick box] |
| **Standard-risk adults** | [Tick box] | [Tick box] |
| **High-risk adults** | [Tick box] | [Tick box] |
| **Adolescents and young adults** | [Tick box] | [Tick box] |
| **Elderly patients** | [Tick box] | [Tick box] |
| **Other ___________________** | [Tick box] | [Tick box] |

# Marker identification for later MRD screening

The following questions ask about the initial identification of markers for later MRD testing (the leukaemia-associated phenotype for flow cytometry or clonal re-arrangements for molecular PCR).

These questions relate to your caseload of **adults** with **B-precursor ALL** whom you treated in **first line** in the past 12 months.

Q7a. In how many of your patients did you request the identification of markers for later MRD?

*Please select one option for each group of patients. If you answered “some”, please indicate the number of patients for whom you requested the identification of markers for later MRD testing.*

|  | Adults with Ph− disease | | Adults with Ph+ disease | |
| --- | --- | --- | --- | --- |
| *Number of patients with B-precursor ALL* |  |  |  |  |
| **All my patients** | [Radio button] |  | [Radio button] |  |
| **Some of my patients**  Number of patients | [Radio button] |  | [Radio button] |  |
| **None of my patients** | [Radio button] |  | [Radio button] |  |

These questions relate to your caseload of **adults** with **B-precursor ALL** whom you treated in **first line**.

Q7b. Please indicate why you did not request the identification of markers for later MRD testing for all your patients.

*Please select all options that apply for each group of patients. If you select “other”, please state the reason.*

|  | Adults with Ph− disease | Adults with Ph+ disease |
| --- | --- | --- |
| **No bone marrow or blood sample available** | [Tick box] | [Tick box] |
| **Poor physical status of the patient** | [Tick box] | [Tick box] |
| **The protocol/trial does not specify that MRD testing is required** | [Tick box] | [Tick box] |
| **The treatment decision is not guided by MRD** | [Tick box] | [Tick box] |
| **MRD testing is not reimbursed** | [Tick box] | [Tick box] |
| **Other _________________** | [Tick box] | [Tick box] |

The following questions ask about the initial identification of markers for later MRD testing.

These questions relate to your caseload of **adults** with **B-precursor ALL** whom you treated in **first line**.

Q8a. When do you usually request the identification of markers for later MRD testing?

*Please select one option for each group of patients.*

|  | Adults with Ph− disease | Adults with Ph+ disease |
| --- | --- | --- |
| At the time of the diagnostic work-up prior to the start of induction therapy | [Radio button] | [Radio button] |
| At a different time | [Radio button] | [Radio button] |

Q8b. Please state when the testing is conducted.

|  |
| --- |
|  |

Q8c. Why was the identification of markers for later MRD testing conducted at a different time point to the diagnostic work-up?

|  |
| --- |
|  |

The following questions relate to the methods of testing that are available to you for conducting the identification of markers for later MRD testing. The methods described are molecular polymerase chain reaction (PCR) and flow cytometry.

Q9. In your clinical practice, what methods are available for the identification of markers for later MRD testing for patients?

*Please select one option.*

| **Only molecular PCR is available** | [Radio button] |
| --- | --- |
| **Only flow cytometry is available** | [Radio button] |
| **Both molecular PCR and flow cytometry are available** | [Radio button] |

*Q10 was removed after pilot phase, but question numbering was maintained for programming*

Q11. What type of laboratory did you use for the identification of markers for later MRD testing?

Please distinguish between a central laboratory (e.g. serving multiple institutions, potentially a reference centre for a protocol or trial), and a local laboratory (e.g. within your institution).

*Please select all options that apply for each group of patients.*

|  | Adults with Ph− disease | Adults with Ph+ disease |
| --- | --- | --- |
| **PCR marker identification in central laboratory** | [Tick box] | [Tick box] |
| **PCR marker identification in local laboratory** | [Tick box] | [Tick box] |
| **Flow cytometry marker identification in central laboratory** | [Tick box] | [Tick box] |
| **Flow cytometry marker identification in local laboratory** | [Tick box] | [Tick box] |

*Q12 was removed after pilot phase, but question numbering was maintained for programming*

Q13a. It may not always be possible to determine suitable markers using your preferred method of identification. Of the patients you treated in the past 12 months, how many had markers determined by each method?

*Please indicate number of patients with markers for future MRD testing established by each method over the past 12 months, and those unable to establish for each group of patients. If a patient had markers determined by both methods, please count in both rows.*

|  | Adults with Ph− disease | | Adults with Ph+ disease | | |
| --- | --- | --- | --- | --- | --- |
| Number of patients treated |  |  |  | |  |
| Number of patients with identification of MRD markers requested |  |  |  |  | |
| **Clonal re-arrangements identified by PCR** |  | % |  | % | |
| **Leukaemia-associated phenotype determined by flow cytometry** |  | % |  | % | |
| **No leukaemia-associated phenotype or clonal re-arrangements determined** |  | % |  | % | |

*Q14 was removed after pilot phase, but question numbering was maintained for programming*

# First MRD test in first-line treatment

The questions relate to the first MRD test that is used to determine risk and establish the treatment plan for a given patient (referred to as the “**prognostic MRD test**”). All questions relate to your caseload of **adults** with **B-precursor ALL** who have achieved haematological CR for the first time with **first-line** treatment over the past 12 months.

Please provide the following information about the timing of the **prognostic MRD test.**

Q15a. Please report the **earliest time point** when testing was conducted

*Please select either weeks or blocks of chemotherapy to provide the time point for each group of patients.*

| Adults with Ph− disease | | |
| --- | --- | --- |
| Time point stated in | |  |
| [Radio button] | Weeks | Test conducted _____ weeks after start of induction therapy |
| [Radio button] | Blocks of chemotherapy | Test conducted _____ blocks after start of induction therapy |
|  |  | Duration of block: ____ weeks |
| Adults with Ph+ disease | |  |
| Time point stated in | |  |
| [Radio button] | Weeks | Test conducted _____ weeks after start of induction therapy |
| [Radio button] | Blocks of chemotherapy | Test conducted _____ blocks after start of induction therapy |
|  |  | Duration of block: ____ weeks |

Q15b. Please report the **latest time point** when testing was conducted

*Please select either weeks or blocks of chemotherapy to provide the time point for each group of patients.*

| Adults with Ph− disease | | |
| --- | --- | --- |
| Time point stated in | |  |
| [Radio button] | Weeks | Test conducted _____ weeks after start of induction therapy |
| [Radio button] | Blocks of chemotherapy | Test conducted _____ blocks after start of induction therapy |
|  |  | Duration of block: ____ weeks |
| Adults with Ph+ disease | |  |
| Time point stated in | |  |
| [Radio button] | Weeks | Test conducted _____ weeks after start of induction therapy |
| [Radio button] | Blocks of chemotherapy | Test conducted _____ blocks after start of induction therapy |
|  |  | Duration of block: ____ weeks |

Q15c. Please report the **most common time point** when testing was conducted

*Please select either weeks or blocks of chemotherapy to provide the time point for each group of patients.*

| Adults with Ph− disease | | |
| --- | --- | --- |
| Time point stated in | |  |
| [Radio button] | Weeks | Test conducted _____ weeks after start of induction therapy |
| [Radio button] | Blocks of chemotherapy | Test conducted _____ blocks after start of induction therapy |
|  |  | Duration of block: ____ weeks |
| Adults with Ph+ disease | |  |
| Time point stated in | |  |
| [Radio button] | Weeks | Test conducted _____ weeks after start of induction therapy |
| [Radio button] | Blocks of chemotherapy | Test conducted _____ blocks after start of induction therapy |
|  |  | Duration of block: ____ weeks |

The questions relate to the first MRD test that is used to determine risk and establish the treatment plan for a given patient (referred to as the “prognostic MRD test”). All questions relate to your caseload of **adults** with **B-precursor ALL** who have achieved haematological CR for the first time with **first-line** treatment over the past 12 months.

Q15d. Which factor most influenced the timing of the **prognostic MRD test**?

*Please select one option for each group of patients.*

|  | Adults with Ph− disease | Adults with Ph+ disease |
| --- | --- | --- |
| **Determined by clinical circumstances** | [Radio button] | [Radio button] |
| **Determined by the protocol/trial I follow** | [Radio button] | [Radio button] |
| **Other ___________** | [Radio button] | [Radio button] |

The following questions relate to the protocol/trials you indicated were the ones you most commonly used in determining the treatment pathway for adults with B-precursor ALL whom you have treated in first line.

Q16a. For patients treated according to those protocols/trials, have you conducted the **prognostic MRD test** at a different time point than specified in the protocol/trial?

*Please select all that apply for each group of patients.*

|  | Adults with Ph− disease | Adults with Ph+ disease |
| --- | --- | --- |
| **Most common protocol/trial** |  |  |
| **Yes, earlier than the protocol/trial specified** | [Tick box] | [Tick box] |
| **Yes, later than the protocol/trial specified** | [Tick box] | [Tick box] |
| **No** | [Radio button] | [Radio button] |

Q16b. Which factors influenced the decision to test at a different time point?

*Please select all options that apply for each group of patients, and indicate the most common factor. If you select “other”, please state the reason.*

|  | Adults with Ph− disease | | Adults with Ph+ disease | |
| --- | --- | --- | --- | --- |
|  | Factor used | Most common | Factor used | Most common |
| **Insufficient bone marrow at time of scheduled test** | [Tick box] | [Radio button] | [Tick box] | [Radio button] |
| **Peripheral blood count was low at time of scheduled test** | [Tick box] | [Radio button] | [Tick box] | [Radio button] |
| **Patient had not achieved CR at time of scheduled test** | [Tick box] | [Radio button] | [Tick box] | [Radio button] |
| **Patient was unfit to continue current planned treatment at time of scheduled test** | [Tick box] | [Radio button] | [Tick box] | [Radio button] |
| **Patient was elderly** | [Tick box] | [Radio button] | [Tick box] | [Radio button] |
| **Patient’s physical status was poor** | [Tick box] | [Radio button] | [Tick box] | [Radio button] |
| **Other _____** | [Tick box] | [Radio button] | [Tick box] | [Radio button] |

The following questions relate to all adults with B-precursor ALL whom you have treated in first line, regardless of whether the treatment was guided by a protocol/trial.

Q16c. In your clinical practice, what methods are available for the MRD prognostic test for patients in first-line treatment?

*Please select one option.*

|  | Patients treated in first line |
| --- | --- |
| **Only molecular PCR is available** | [Radio button] |
| **Only flow cytometry is available** | [Radio button] |
| **Both molecular PCR and flow cytometry are available** | [Radio button] |

Q16d. How often did you use each method in the past 12 months for conducting the MRD prognostic test in patients in first-line treatment?

*Please state the number of patients you tested by each method for each group of patients over the past 12 months (options can sum to more than 100%, if samples from the same patient were sent for testing by each method).*

|  | Adults with Ph− disease | | Adults with Ph+ disease | |
| --- | --- | --- | --- | --- |
| Total number of patients in caseload |  |  |  |  |
| **Total number of patients with a sample sent for MRD prognostic test** |  |  |  |  |
| **Number of patients with sample sent for testing using PCR** |  | % |  | % |
| **Number of patients with sample sent for testing using flow cytometry** |  | % |  | % |

Q16e. What type of laboratory did you use to conduct MRD prognostic tests by PCR or flow cytometry, respectively?

Please distinguish between a central laboratory (e.g. serving multiple institutions, potentially a reference centre for a protocol or trial), and a local laboratory (e.g. within your institution).

*Please select all options that apply for each group of patients.*

|  | Adults with Ph− disease | | Adults with Ph+ disease | |
| --- | --- | --- | --- | --- |
|  | PCR | **Flow cytometry** | PCR | **Flow cytometry** |
| Local | [Tick box] | [Tick box] | [Tick box] | [Tick box] |
| Central | [Tick box] | [Tick box] | [Tick box] | [Tick box] |

Q16f. How do you determine which laboratory to use for the MRD prognostic testing?

*Please select all options that apply for each method.*

|  | PCR | | Flow cytometry | |
| --- | --- | --- | --- | --- |
|  | Central | Local | Central | Local |
| **It is the reference laboratory specified in the treatment protocol/trial I most commonly follow for this group of patients** | [Tick box] | [Tick box] | [Tick box] | [Tick box] |
| **There are local financial arrangements with the laboratory** | [Tick box] | [Tick box] | [Tick box] | [Tick box] |
| **The laboratory is close to my centre** | [Tick box] | [Tick box] | [Tick box] | [Tick box] |
| **Other ______________** | [Tick box] | [Tick box] | [Tick box] | [Tick box] |

Q16g. Do the laboratory(s) you use follow national or international standardized protocols for MRD determination (e.g. from the EuroFlow or EuroMRD consortiums)?

*Please select the option that applies for each method*.

|  | PCR | | Flow cytometry | |
| --- | --- | --- | --- | --- |
|  | Central | Local | Central | Local |
| **Yes** | [Radio button] | [Radio button] | [Radio button] | [Radio button] |
| **No** | [Radio button] | [Radio button] | [Radio button] | [Radio button] |
| **Not sure** | [Radio button] | [Radio button] | [Radio button] | [Radio button] |

Q17. What was the average number of days from taking a sample for the **MRD prognostic test** to receiving the test results?

*Please state the number of days for each group of patients and each method.*

|  | Adults with Ph− disease | | | | Adults with Ph+ disease | | | |
| --- | --- | --- | --- | --- | --- | --- | --- | --- |
|  | PCR | | Flow cytometry | | PCR | | Flow cytometry | |
|  | Local | Central | Local | Central | Local | Central | Local | Central |
| **Number of days** | ____ days | ____ days | ____ days | ____ days | ____ days | ____ days | ____ days | ____ days |

The following question relates to the results of the MRD prognostic test.

Q18a. What threshold do you most commonly associate with establishing whether a patient has MRD− status?

*Please select one option only for each method of MRD testing. If you select “other”, please state the threshold you use.*

|  | Results based on PCR | Results based on flow cytometry |
| --- | --- | --- |
| **MRD− if % leukemic cells <10^−2^** | [Radio button] | [Radio button] |
| **MRD− if % leukemic cells <10^−3^** | [Radio button] | [Radio button] |
| **MRD− if % leukemic cells <10^−4^** | [Radio button] | [Radio button] |
| **MRD− if % leukemic cells <10^−5^** | [Radio button] | [Radio button] |
| **No detectable leukemic cells** | [Radio button] | [Radio button] |
| **Other _________** | [Radio button] | [Radio button] |

Q19. When you have received the results of the **MRD prognostic test**, what was the most common format for the results?

*Please select one option only for each testing method.*

| Results based on PCR | | Results based on flow cytometry | |
| --- | --- | --- | --- |
| **% leukemic cells is reported** | | | |
| Yes | [Radio button] | Yes | [Radio button] |
| No | [Radio button] | No | [Radio button] |
| **Associated threshold of leukemic cells is reported** | | | |
| Yes | [Radio button] | Yes | [Radio button] |
| No | [Radio button] | No | [Radio button] |
| **Who determines the MRD status?** | | | |
| Laboratory | [Radio button] | Laboratory | [Radio button] |
| The protocol/trial I most commonly follow | [Radio button] | The protocol/trial I most commonly follow | [Radio button] |
| Determined by another protocol/trial | [Radio button] | Determined by another protocol/trial | [Radio button] |
| I determine the MRD status | [Radio button] | I determine the MRD status | [Radio button] |

The following questions relate to the results of the prognostic MRD test and how that has influenced your treatment decisions. The questions should be answered in relation to your caseload of adults with B-precursor ALL whom you have treated in first line over the past 12 months.

Q20a. Were your treatment decisions guided by the results of the MRD prognostic test?

*Please select one option only for each group of patients.*

|  | Adults with Ph− disease | Adults with Ph+ disease |
| --- | --- | --- |
| **Yes, it is my decision on appropriate treatment based on the results** | [Radio button] | [Radio button] |
| **Yes, they were protocol/trial-stipulated treatment decisions based on the results** | [Radio button] | [Radio button] |
| **No, treatment decisions were not guided by results** | [Radio button] | [Radio button] |

Q20b. What treatment decisions were determined by the results of the prognostic MRD test?

*Please select all options that apply for each group of patients.*

|  | Adults with Ph− disease | | Adults with Ph+ disease | |
| --- | --- | --- | --- | --- |
|  | If MRD− | If MRD+ | If MRD− | If MRD+ |
| **Start maintenance/consolidation treatment** | [Tick box] | [Tick box] | [Tick box] | [Tick box] |
| **Start treatment intensification** | [Tick box] | [Tick box] | [Tick box] | [Tick box] |
| **Decide suitability for SCT** | [Tick box] | [Tick box] | [Tick box] | [Tick box] |
| **Other ______** | [Tick box] | [Tick box] | [Tick box] | [Tick box] |

Q20c. Do you usually have results from more than one source (laboratory and/or methodology) for the MRD prognostic test for the same patient?

*Please select one option only for each group of patients.*

|  | Adults with Ph− disease | Adults with Ph+ disease |
| --- | --- | --- |
| **Yes** | [Radio button] | [Radio button] |
| **No** | [Radio button] | [Radio button] |

Q20d. If you have the results from more than one source for the same patient, which results do you use for your treatment decision-making?

*Please select one option.*

|  | Adults with Ph− disease | Adults with Ph+ disease |
| --- | --- | --- |
| **PCR result from local lab** | [Radio button] | [Radio button] |
| **PCR result from central lab** | [Radio button] | [Radio button] |
| **Flow cytometry result from local lab** | [Radio button] | [Radio button] |
| **Flow cytometry result from central lab** | [Radio button] | [Radio button] |
| **Combination of several results** | [Radio button] | [Radio button] |

Q20e. How do you determine which result you use to make treatment decisions?

*For each patient group, please select all that reasons that apply to you and your clinic.*

|  | Adults with Ph− disease | Adults with Ph+ disease |
| --- | --- | --- |
| **Stipulated in the treatment protocol/trial I most commonly follow for this group of patients** | [Tick box] | [Tick box] |
| **Other ______________** | [Tick box] | [Tick box] |

Q20f. If you use a combination of several results for the same patient, please explain the circumstances and how you make your decisions?

________________________________________

# Post-CR MRD tests in first-line treatment

The following questions explore the tests used to monitor MRD status after a patient has achieved CR for the first time (referred to as **post-CR MRD tests**).

The questions should be answered in relation to your caseload of adults with B-precursor ALL whom you treat in first line.

The time period of interest is the 12 months immediately after the prognostic MRD test, which occurs between CR1 and SCT (if relevant); the responses should include all patients treated in the last few years, ideally focusing on patients treated in the last 12 months, even though some may not yet have been followed up for 12 months.

Q21a. For patients who have achieved complete haematological remission for the first time (i.e., CR1), how many MRD tests did you conduct over the subsequent 12 months?

This should exclude the identification of markers and the prognostic MRD test immediately after CR; it should also exclude any tests conducted after SCT.

*Please include all relevant tests, including any specified in the protocol/trial and any additional tests, for each group of patients. If tests were specified in the protocol/trial but not conducted, please exclude them from the total.*

|  | Adults with Ph− disease | | Adults with Ph+ disease | |
| --- | --- | --- | --- | --- |
| MRD status based on first prognostic test | MRD+ | MRD- | MRD+ | MRD- |
| **Lowest number of tests conducted for an individual patient** |  |  |  |  |
| **Highest number of tests conducted for an individual patient** |  |  |  |  |
| **Most common number of tests conducted for an individual patient** |  |  |  |  |

Q21a2. How frequently did you usually test for MRD in patients in CR1?

|  |  |  |  |  |
| --- | --- | --- | --- | --- |
|  | Adults with Ph− disease | | Adults with Ph+ disease | |
| MRD status in prognostic MRD test | MRD+ | MRD- | MRD+ | MRD- |
| **Interval** | I conduct an MRD test every ________ months | I conduct an MRD test every ________ months | I conduct an MRD test every ________ months | I conduct an MRD test every ________ months |

Q21b. Which factor most influenced the number of **post-CR MRD tests**?

*Please select one option only for each group of patients.*

|  | Adults with Ph− disease | Adults with Ph+ disease |
| --- | --- | --- |
| **Clinical circumstances determine frequency** | [Radio button] | [Radio button] |
| **Protocol/trial-stipulated number** | [Radio button] | [Radio button] |
| **Other __________** | [Radio button] | [Radio button] |

The following question relates only to the adults with B-precursor ALL whom you treated in first line with your most commonly used protocol/trial (as previously defined) over the past 12 months.

Q22. Which of the following MRD testing time points are included in the protocol/trial? This question relates to the period post CR1 and before SCT (if relevant).

*Please select all options that apply for each group of patients.* *If you select “other”, please state the time point.*

|  | Adults with Ph− disease | Adults with Ph+ disease |
| --- | --- | --- |
| **Most common protocol/trial** |  |  |
| **Test after the start of induction (i.e., prognostic MRD test)** | [Tick box] | [Tick box] |
| **Test after fixed number of cycles of consolidation therapy** | [Tick box] | [Tick box] |
| **Test after treatment intensification** | [Tick box] | [Tick box] |
| **Test before SCT** | [Tick box] | [Tick box] |
| **Test during maintenance** | [Tick box] | [Tick box] |
| **Others __________** | [Tick box] | [Tick box] |

Q23. Did you conduct the number of MRD tests specified in the protocol/trial in all patients?

|  | Adults with Ph− disease | | Adults with Ph+ disease | |
| --- | --- | --- | --- | --- |
| MRD status in prognostic MRD test | MRD+ | MRD- | MRD+ | MRD- |
| Yes, I conducted the same number as specified in all patients | [Radio button] | [Radio button] | [Radio button] | [Radio button] |
| No, in some patients I conducted more than the number specified | [Tick box] | [Tick box] | [Tick box] | [Tick box] |
| No, in some patients I conducted fewer than the number specified | [Tick box] | [Tick box] | [Tick box] | [Tick box] |

Q24a. If you conducted a different number of tests from that specified in the protocol/trial, which factors influenced this?

*Please select all options that apply for each group of patients, and indicate the most common reason. If you select “other”, please state the reason.*

|  | Adults with Ph− disease | | Adults with Ph+ disease | |
| --- | --- | --- | --- | --- |
|  | Factor used | Most common | Factor used | Most common |
| **Insufficient bone marrow at time of scheduled test** | [Tick box] | [Radio button] | [Tick box] | [Radio button] |
| **Peripheral blood count was low at time of scheduled test** | [Tick box] | [Radio button] | [Tick box] | [Radio button] |
| **Patient was unfit to continue current planned treatment** | [Tick box] | [Radio button] | [Tick box] | [Radio button] |
| **Patient was elderly** | [Tick box] | [Radio button] | [Tick box] | [Radio button] |
| **Patient’s physical status was poor** | [Tick box] | [Radio button] | [Tick box] | [Radio button] |
| **Other ___________** | [Tick box] | [Radio button] | [Tick box] | [Radio button] |

Q24b. Where do you usually conduct post-CR MRD tests? Please indicate which results are used for treatment decision making.

*Please select all options that apply for each group of patients.*

|  | Adults with Ph− disease | | Adults with Ph+ disease | |
| --- | --- | --- | --- | --- |
|  | Tests to monitor MRD status | Results used for treatment decision-making | Tests to monitor MRD status | Results used for treatment decision-making |
| **PCR in central lab** | [Tick box] | [Tick box] | [Tick box] | [Tick box] |
| **PCR in local lab** | [Tick box] | [Tick box] | [Tick box] | [Tick box] |
| **Flow cytometry in central lab** | [Tick box] | [Tick box] | [Tick box] | [Tick box] |
| **Flow cytometry in local lab** | [Tick box] | [Tick box] | [Tick box] | [Tick box] |

Some patients are classified as MRD+ based on the results of the prognostic test conducted after they achieve CR1, but are later found to have MRD− status. The following questions relate to these patients.

Q25a. How would the treatment pathway for the patient change as a result of the MRD– status?

*Please select one option only for each group of patients.*

|  | **Adults with Ph− disease** | **Adults with Ph+ disease** |
| --- | --- | --- |
| **Treatment pathway would not change** | [Radio button] | [Radio button] |
| **Planned SCT would not be carried out** | [Tick box] | [Tick box] |
| **SCT would be postponed** | [Tick box] | [Tick box] |
| **Chemotherapy regimen would be changed** | [Tick box] | [Tick box] |
| **Chemotherapy would be stopped** | [Tick box] | [Tick box] |
| **Other ___________** | [Tick box] | [Tick box] |

Q25b. Would you change the number of MRD tests, from that planned for a patient who was MRD+?

*Please select one option only for each group of patients.*

|  | Adults with Ph− disease | Adults with Ph+ disease |
| --- | --- | --- |
| **No, test as originally planned** | [Radio button] | [Radio button] |
| Yes, increase the number of planned MRD tests | [Radio button] | [Radio button] |
| Yes, decrease the number of planned MRD tests | [Radio button] | [Radio button] |

Q26. If you change the treatment a patient is given (e.g., a different regimen or a switch to SCT), do you test for MRD at this time?

*Please select one option only for each group of patients.*

|  | Adults with Ph− disease | Adults with Ph+ disease |
| --- | --- | --- |
| **Yes** | [Radio button] | [Radio button] |
| **No** | [Radio button] | [Radio button] |
| **Determined by clinical circumstances** | [Radio button] | [Radio button] |

# MRD testing for patients with prior relapse

The following questions focus on MRD testing in patients who have experienced a relapse but have subsequently achieved a second (or later) complete haematological remission (referred to as patients in CR2+).

The questions should be answered in relation to your caseload of adults with B-precursor ALL whom you have treated over the past 12 months.

Q27. What are your main reasons for conducting MRD testing in patients in CR2+?

|  |
| --- |
|  |

Q28a. Do you follow a protocol or trial for the treatment of these patients?

*Please select one option only for each group of patients.*

|  | Adults with Ph− disease | Adults with Ph+ disease |
| --- | --- | --- |
| **Yes** | [Radio button] | [Radio button] |
| **No** | [Radio button] | [Radio button] |

Q28b. Is a MRD testing schedule for patients in CR2+ stipulated in the protocol/trial?

*Please select one option only for each group of patients.*

|  | Adults with Ph− disease | Adults with Ph+ disease |
| --- | --- | --- |
| **Yes** | [Radio button] | [Radio button] |
| **No** | [Radio button] | [Radio button] |

Q28c. How frequently did you usually test for MRD in patients in CR2+ assuming patients did not experience a further relapse?

*Please select one option only for each group of patients.*

|  | Adults with Ph− disease | Adults with Ph+ disease |
| --- | --- | --- |
| **Interval** | I conduct an MRD test every ________ months | I conduct an MRD test every ________ months |

Q27c. What factors most influence your MRD testing frequency in patients with CR2+?

*Please select all options that apply.*

|  | Adults with Ph− disease | Adults with Ph+ disease |
| --- | --- | --- |
| **MRD test does not have prognostic value in patient with CR2+** | [Tick box] | [Tick box] |
| **MRD tests are not funded for patients in CR2+** | [Tick box] | [Tick box] |
| **Other ________________** | [Tick box] | [Tick box] |

Q27d. For patients in CR2+, where do you usually conduct the tests used to monitor MRD status? Please indicate which results are used for treatment decision making.

*Please select all options that apply for each group of patients.*

|  | Adults with Ph− disease | | Adults with Ph+ disease | |
| --- | --- | --- | --- | --- |
|  | Tests to monitor MRD status | Results used for treatment decision-making | Tests to monitor MRD status | Results used for treatment decision-making |
| **PCR in central lab** | [Tick box] | [Tick box] | [Tick box] | [Tick box] |
| **PCR in local lab** | [Tick box] | [Tick box] | [Tick box] | [Tick box] |
| **Flow cytometry in central lab** | [Tick box] | [Tick box] | [Tick box] | [Tick box] |
| **Flow cytometry in local lab** | [Tick box] | [Tick box] | [Tick box] | [Tick box] |

# MRD funding

Questions were included on this topic: this data is not described in the current publication

# Healthcare resource use

Questions were included on this topic: this data is not described in the current publication

# Future of MRD testing

The following section asks you about your thoughts on the future of MRD testing for adults with B-precursor ALL.

Q38. Over the next 6–12 months, do you think that emerging treatments will change the pattern of MRD testing for first-line treatment of adults with B-precursor ALL?

| **Yes** | [Radio button] |
| --- | --- |
| **No** | [Radio button] |

Q39. How do you see MRD testing evolving?

|  |
| --- |

Q40. Do you anticipate any additional restrictions being imposed on MRD testing in the next 6–12 months?

| **Yes** | [Radio button] |
| --- | --- |
| **No** | [Radio button] |

Q42. If yes, what kind of restrictions?

|  |
| --- |
