# Supplementary material for: Testing for minimal residual disease in adults with acute lymphoblastic leukemia in Europe: a clinician survey
Source: BMC Cancer. 2018 Nov 12;18:1100. doi: 10.1186/s12885-018-5002-5 (PMC6233570; doi:10.1186/s12885-018-5002-5)
Supplement: Supplementary file 3 — Supplementary tables. (DOCX 48 kb) [file 12885_2018_5002_MOESM3_ESM.docx]

Additional file 3

**Table S1** Demographics of participants

|  | Pooled across countries (n = 103) | France (n = 20) | Germany (n = 20) | Italy (n = 20) | Spain (n = 23) | UK (n = 20) |
| --- | --- | --- | --- | --- | --- | --- |
| Clinical role Hematologist Hemato-oncologist | 54 (52%) 49 (48%) | 15 (75%)  5 (25%) | 0 20 (100%) | 15 (75%) 5 (25%) | 14 (61%) 9 (39%) | 10 (50%) 10 (50%) |
| Years of experience in treating ALL since completion of specialty training Mean (SD) Median (IQR) Range: min–max | 16 (6) 15 (11–20) 6–32 | 18 (5) 18 (15–20) 7–28 | 14 (5) 15 (12–18) 6–20 | 14 (8) 10 (9–21) 8–32 | 17 (5) 16 (14–22) 6–27 | 15 (5) 15 (12–20) 7–26 |
| Type of center (n [%]) University hospital Community/urban/general hospital Cancer hospital or specialist oncology center Specialist hematology center | 68 (66%) 17 (17%) 15 (15%) 3 (3%) | 15 (75%) 3 (15%) 2 (10%) 0 | 7 (35%) 2 (10%) 9 (45%) 2 (10%) | 11 (55%)  6 (30%) 2 (10%) 1 (5%) | 21 (91%) 2 (9%) 0 0 | 14 (70%) 4 (20%) 2 (10%) 0 |
| Participation of institute in research conducted by European ALL study group or other registered trials Yes No | 80 (78%) 23 (22%) | 18 (90%) 2 (10%) | 13 (65%) 7 (35%) | 19 (95%) 1 (5%) | 15 (65%) 8 (35%) | 15 (75%) 5 (25%) |
| Availability of autologous or allogeneic SCT at physician’s center Yes No | 99 (96%) 4 (4%) | 20 (100%) 0 | 20 (100%) 0 | 19 (95%) 1 (5%) | 23 (100%) 0 | 17 (85%) 3 (15%) |

ALL, acute lymphoblastic leukemia; IQR, interquartile range, SCT, stem cell transplantation; SD, standard deviation

**Table S3** MRD testing and stratification in key European treatment protocols

| Country | Treatment protocol (source) | Key MRD testing time points and recommendations |
| --- | --- | --- |
| Germany^a^ | GMALL 06/99 GMALL 07/03[8, 21, 32] | Assessed during induction 1 (day 11), after induction 1 (day 24 or 26), after induction 2 (day 46), before consolidation 1 (day 71), after consolidation 1 (week 16), and at further time points during consolidation treatment and follow-up; essential time points are day 71 and week 16 High-risk MRD (>10^−4^) after first consolidation (week 16): HSCT in first CR Low-risk MRD after induction and first consolidation: no maintenance therapy  Intermediate-risk MRD (i.e., inconclusive MRD course or technical problems): intensified maintenance therapy |
| France^b^ | GRAALL 02/2005[20] | MRD ≥10^−2^ after induction or MRD ≥10^−2^ after 3 consolidation blocks: allogeneic HSCT in first hematological CR |
| Italy | GIMEMA ALL 0904[25, 26] | Assessed during induction at days 35 and 50, and at the end of consolidation; essential time point is during induction therapy |
|  | GIMEMA LAL 1913[33] | Assessed during induction/consolidation at weeks 4, 10, 16 and 22  MRD negative (MRD <10^−4^ at 2^nd^ and 3^rd^ time points, negative at 4^th^ time point): proceed to maintenance therapy  MRD positive (MRD ≥10^−4^ at 2^nd^ and 3^rd^ time points, positive at 4^th^ time point): proceed to allogeneic HSCT if possible, or autologous HSCT followed by maintenance therapy if allogeneic HSCT is not possible |
| Spain | PETHEMA ALL-AR-03[18] | Assessed end of induction (weeks 5–6), end of consolidation 3 (weeks 16–18) Flow-MRD level ≥5 × 10^−4^ at end of consolidation: Allogeneic HSCT |
|  | PETHEMA LAL-ER/2011[34] | Assessed end of induction 1 (day 28):  If CR and MRD <10^−4^ post induction 1: proceed to consolidation: if MRD <10^−5^ after consolidation proceed to late consolidation and maintenance; if MRD ≥10^−5^, proceed to HSCT. If CR and MRD ≥10^−4^ post induction 1: proceed to induction 2 and consolidation; if MRD <10^−4^ post consolidation I, proceed to HSCT; if MRD ≥10^−4^ post consolidation I, proceed to HSCT or experimental treatment. |
|  | PETHEMA LAL PH-2008[35] | Assessed at time of CR, at end of consolidation, pre-transplantation, and post-transplantation (every 1.5 months during the first two years, and every 3 months thereafter).  If MRD is detected pre-transplantation (>10^−4^): treatment with dasatinib  If MRD is detected post-transplantation: treatment with imatinib. |
| UK | UKALL14[22] | Assessed end of Phase 1 induction (after week 4); end of Phase 2 induction (after week 8); after transplantation (if applicable) High-risk (MRD at end of induction therapy): eligible for allogeneic HSCT Low-risk (absence of MRD at end of induction therapy): consolidation and maintenance chemotherapy, not allogeneic HSCT |

^a^ The more recent GMALL 08/2013 was selected as the most common choice: however this protocol is not publicly available. ^a^ The more recent GRAALL 2014 and ALL GRAALLPHAG06/EWALL-PH–01 were selected as the most common choice: however these protocols are not publicly available.
CR, complete remission; HSCT, hematopoietic stem cell transplantation; MRD, minimal residual disease

**Table S4** Most common time point reported for the prognostic MRD test (weeks from start of induction therapy)

|  | Adults with Ph− disease | | Adults with Ph+ disease | |
| --- | --- | --- | --- | --- |
|  | Mean (SD) | Median (IQR) | Mean (SD) | Median (IQR) |
| France | 11 (27) | 5 (4–6) | 8 (14) | 5 (4–6) |
| Germany | 6 (5) | 4 (3–8) | 7 (6) | 4 (3–10) |
| Italy | 11 (16) | 4 (4–12) | 10 (15) | 4 (4–8) |
| Spain | 6 (3) | 4 (4–8) | 6 (4) | 4 (4–8) |
| UK | 7 (5) | 5 (4–8) | 7 (5) | 4 (4–8) |
| All countries | 8 (14) | 4 (4–8) | 7 (10) | 4 (4–8) |

IQR, interquartile range; MRD, minimal residual disease; Ph, Philadelphia chromosome translocation; SD, standard deviation

**Table S5** Laboratory used for the prognostic MRD test

| Percentage of clinicians | Adults with Ph− disease | | | | Adults with Ph+ disease | | | |
| --- | --- | --- | --- | --- | --- | --- | --- | --- |
|  | n | Central | Local | Both | n | Central | Local | Both |
| France | 19 | 58% | 11% | 32% | 19 | 58% | 26% | 16% |
| Germany | 19 | 53% | 16% | 32% | 16 | 62% | 13% | 25% |
| Italy | 14 | 14% | 14% | 71% | 17 | 24% | 29% | 47% |
| Spain | 21 | 29% | 48% | 24% | 19 | 26% | 63% | 11% |
| UK | 19 | 47% | 26% | 26% | 18 | 44% | 33% | 22% |
| All countries | 92 | 41% | 24% | 35% | 89 | 43% | 34% | 24% |

MRD, minimal residual disease; N/A, not applicable; Ph-, Philadelphia chromosome translocation negative; Ph+, Philadelphia chromosome translocation positive

**Table S6** Treatment decisions based on the outcomes of the prognostic MRD test

| Type of treatment decision (pooled data across countries) | Number of responding physicians (%) | | | |
| --- | --- | --- | --- | --- |
|  | Adults with Ph– disease (n=87) | | Adults with Ph+ disease (n=87) | |
|  | MRD- | MRD+ | MRD- | MRD+ |
| Start maintenance/consolidation treatment | 71 (82%) | 22 (25%) | 63 (72%) | 26 (30%) |
| Start treatment intensification | 23 (26%) | 58 (67%) | 26 (30%) | 53 (61%) |
| Decide suitability for SCT | 24 (28%) | 51 (59%) | 36 (41%) | 54 (62%) |
| Other | 1 (1%) | 0 | 0 | 1 (1%) |

MRD, minimal residual disease; N/A, not applicable; Ph-, Philadelphia chromosome translocation negative; Ph+, Philadelphia chromosome translocation positive; SCT, stem cell transplantation
